# ProtMiscuity: a database of promiscuous proteins

**DOI:** 10.1093/database/baz103

**Published:** 2019-10-24

**Authors:** Ana Julia Velez Rueda, Nicolas Palopoli, Matías Zacarías, Leandro Matías Sommese, Gustavo Parisi

**Affiliations:** Departamento de Ciencia y Tecnología, Universidad Nacional de Quilmes - CONICET, Roque Sáenz Peña 352, Bernal B1876BXD Buenos Aires, Argentina

**Keywords:** promiscuity, proteins, database

## Abstract

Promiscuous behaviour in proteins and enzymes remains a challenging feature to understand the structure–function relationship. Here we present ProtMiscuity, a manually curated online database of proteins showing catalytic promiscuity. ProtMiscuity contains information about canonical and promiscuous activities comprising 88 different reactions in 57 proteins from 40 different organisms. It can be searched or browsed by protein names, organisms and descriptions of canonical and promiscuous reactions. Entries provide information on reaction substrates, products and kinetic parameters, mapping of active sites to sequence and structure and links to external resources with biological and functional annotations. ProtMiscuity could assist in studying the underlying mechanisms of promiscuous reactions by offering a unique and curated collection of experimentally derived data that is otherwise hard to find, retrieve and validate from literature.

## Introduction

Even though protein promiscuity has been extensively studied in the last decades, the term itself is not well-defined yet (1). It has been used to describe several distinct phenomena, and different classification schemes have been proposed (2, 3). Khersonsky and Tawfik (4) described catalytic promiscuity as the capability of an enzyme to catalyze a reaction different than that which the protein has evolved to sustain. From a chemical and functional point of view, catalytic promiscuity was described as the ability of an enzyme to catalyze a secondary reaction at the same active site where its primary activity occurs. This secondary reaction must have a different chemical mechanism, usually described with a different name and involving formation and/or breakage of distinct bonds (5). Similarly, substrate promiscuity has also been used to describe the capacity of the enzyme to perform comparable chemical reactions using different substrates (6). Under this perspective, both catalytic and substrate promiscuous activities generally involve substrates and products lacking physiological or biochemical relevance for the organisms (7, 8). For this reason, the use of ‘promiscuity’ to describe proteins and enzymes with broad specificity to biologically relevant ligands should be avoided. For example, proteins that serve more than one physiological function, often regulatory or structural rather than enzymatic, and in different times or cellular compartments, should be more appropriately categorized as moonlighting proteins. Multiple-substrate binding capacity in several proteins is an evolutionary-derived trait, meaning that evolutionary pressure modulated enzyme evolution to fulfil a given biological task (6).

Besides their many definitions and perspectives, promiscuity is far from being an uncommon phenomenon as previously thought and is increasingly permeating into drug discovery protocols, organic synthesis, pharmacology and biotechnology (9, 10). Multiple cases of catalytic promiscuity have been described, involving different mechanisms (11, 12). For example, metalloenzymes are well known as enhancing their catalytic repertoire by cofactor exchange (13, 14). But also, many non-enzyme proteins were described as promiscuous, capable of catalyzing more than one complex reaction. Such is the case of serum albumins, both human and bovine, that showed very diverse catalytic capabilities (15–17) from Kemp elimination reactions to cross aldol condensations. These and many other interesting cases show the complexity of protein functionality and the need for gathering information that could help to understand the underlying mechanisms and origin of promiscuity, as well as an aid in the development of new tools for prediction (1, 6, 18, 19).

In spite of its biological and biotechnological relevance and the possible impact in diverse areas of research in medicine, drug design, evolutionary biology and bioinformatics, there is still no publicly available collection of scientific evidence on protein promiscuity. Here we present ProtMiscuity, an online database that aims to fill this gap by providing a manually curated dataset of promiscuous enzymes and related biological information. Considering the broad scope of meanings referring to the term ‘promiscuity’, our database only considers examples of catalytic promiscuity, following its definition as proteins sustaining different chemical reactions besides the canonical or biological catalyzed reaction.

## Methods and Results

### Aims of ProtMiscuity

ProtMiscuity is a curated database of promiscuous proteins that aims to centralize experimentally characterized examples of this phenomenon. Among all the different meanings of promiscuity (4, 9), our database focuses on the so-called ‘catalytic promiscuity’, described as the capability of an enzyme to catalyze secondary reactions at an active site that is specialized for a different, primary reaction (20). By organizing our knowledge about this specific type of protein promiscuity, we seek to contribute to several technological achievements, including designing new drugs targeted at known active sites for both biomedical or industrial applications (2), providing guidelines for directed evolution of protein structures and facilitating progress in protein engineering to modulate catalytic functions (10).

### Database implementation

An initial dataset of relevant proteins and associated publications was built through the implementation of web-scraping on PubMed (https://www.ncbi.nlm.nih.gov/pubmed/) and text-mining techniques over this bibliography, using standard libraries in the Python programming language. This collection of putative references to promiscuous proteins was inspected to filter out dubious cases by careful consideration of the available evidence, including data collected manually from related publications and databases. This manual curation process included a critical review of full-text papers with experimental data for each protein and reaction, including the verification of protein sequences and active sites along with annotation mappings from other databases.

The curated dataset was converted and stored as a MySQL relational database. A responsive web interface was built for ProtMiscuity, which provides support for easier navigation and visualization of the database contents on multiple devices. It is implemented in HTML, CSS and JavaScript, with Angular4 and NodeJS. ProtMiscuity is hosted on our server and can be freely accessed at http://ufq.unq.edu.ar/protmiscuity.

### Database contents

A total of 58 proteins with one or more characterized catalytic promiscuous activities are described in the database, involving 2001 different protein chain structures in the PDB (21). These proteins are annotated in ProtMiscuity by their UniProt identifiers (22) and complete name. In its current version, ProtMiscuity covers a total of 88 described chemical reactions in proteins coming from 41 different organisms. Among them, ~68% have only one promiscuous reaction, while 20% of the entries have two and ~6% have more than three promiscuous described activities. Reactions, both promiscuous and canonical, are characterized with information obtained from the literature regarding the chemical description of substrates and products that were used in experimental assays, known *Km* and *kcat* values, active site residues and reaction conditions. Likewise, substrates and products related to each described reaction were linked to the information available in PDB Ligand Expo (23) and PubChem (24) to facilitate the identification of possible ligands by chemical similarity.

In order to provide users with further structural and functional information, each protein is also linked to resources such as the CoDNaS database of conformational diversity (25), KEGG pathways (26), Catalytic Site Atlas annotations (27) and QuickGO terms (28). ProtMiscuity also includes a tutorial section and answers to frequently asked questions to facilitate navigation and use by non-experienced users. All data can be downloaded as a formatted text file. ProtMiscuity will be updated on a regular basis as new evidence becomes available.

ProtMiscuity will be updated periodically. In order to expand its growth, we provide a spreadsheet template that users can download and complete to send feedback about missing entries or specific information about protein promiscuity.

**Figure 1:**
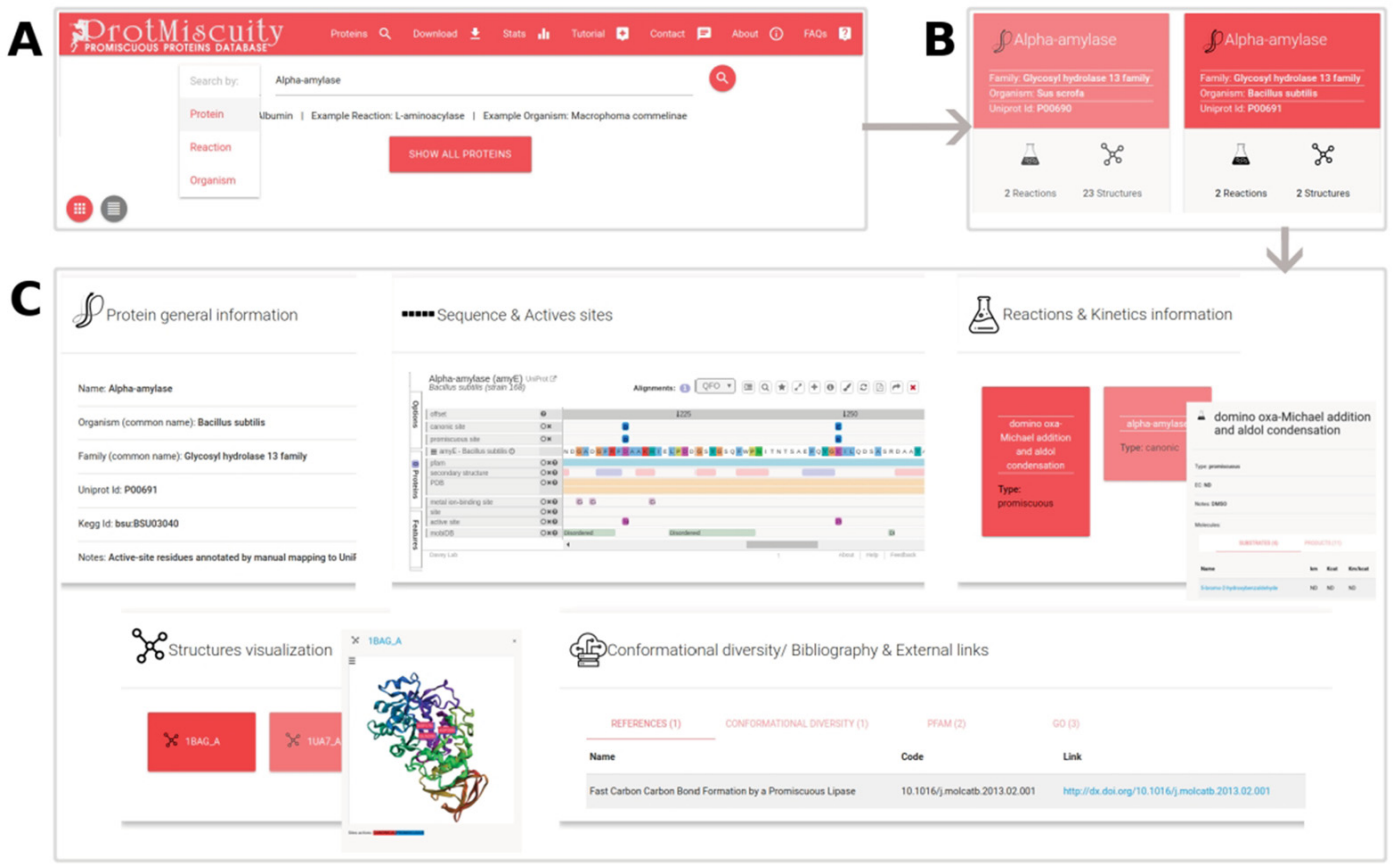
(**A**) Homepage of ProtMiscuity. The database can be searched using protein names, organism and target reaction. In this example, a search for the protein alpha-amylase is performed. (**B**) Results page. It shows all matches to the query term in the form of protein-specific cards. In this example, alpha-amylases from two distinct organisms are retrieved. (**C**) Information page. Clicking on one protein’s card displays all the available information about it, organized in five sections of interest. From top to bottom, left to right: a general description of the protein; the mapping of the canonic and promiscuous active sites, along with other sources of relevant information, on the protein’s sequence; information about canonic and promiscuous activities, with known substrates, products and kinetic parameters (top panel); a visualization of each available structure of the protein, with catalytic sites mapped on it; and examples of conformational diversity, plus links to relevant bibliography and other databases, as separate tabs (bottom panel).

### Database access and user interface

ProtMiscuity can be searched by protein name or UniProt ID, by organism and by the description of canonical or promiscuous activities. An index of proteins is also available that allows browsing the database. A typical query using the protein name retrieves general information about it in the form of browsable cards, including the protein family, source organism, the number of promiscuous and canonical reactions in which it is involved and the number of related structures. Searching with a molecule name or putative substrates/products of catalysis retrieves all proteins linked with the query or with similar molecules (Figure 1). By clicking on a protein, the user is directed to its dedicated page, which displays detailed information on the protein, including its canonic and promiscuous reaction sites mapped onto sequences and known structures using Proviz (29).

## Conclusions

Understanding the origin and mechanisms related with promiscuity may be a key feature for a deeper interpretation of protein function and evolution. Characterization of promiscuous behaviour has broaden the chemical repertoire of enzymatic reactions, uncovering a large number of potential applications in biotechnology and related areas (2, 3, 9, 10). Unfortunately, the lack of a clear and unified description of the different aspects of protein promiscuity makes it hard to recognize examples in the literature.

ProtMiscuity provides a unique and useful resource for exploring new putative catalytic activities and their underlying mechanisms. Inspection of the database shows that catalytic promiscuity is a conserved feature across taxonomic lineages. For example, we found that the AB hydrolase superfamily has the most members in our database (followed closely by tautomerases, both of proven promiscuous behaviours), which are present in several bacteria and fungi, but also in common wheat and pig. In its current version, the database offers 54 curated examples of promiscuous chemical reactions involving ~580 different products and substrates. It is interesting to realize that 12% of the 58 listed proteins have more than two promiscuous reactions, although it is still not clear how these reactions complement each other.

In order to improve annotation and coverage in ProtMiscuity, we welcome feedback from users about new examples of catalytic promiscuity as well as missing entries or information. As the number of entries keeps growing, ProtMiscuity can better help to obtain complete information to develop and test new computational tools for the study and prediction of promiscuous behaviour.

The availability of curated examples as those offered by ProtMiscuity could be important to deepen into conceptual issues in protein promiscuity such as its evolutionary origin and its impact on protein dynamics and chemical versatility (30). Also, curated datasets show alternative cavities, surfaces and amino acid arrangements (31, 32), enabling users to gather data on multiple new catalytic active site descriptions that can improve the design of protein engineering protocols and the discovery of in silico drugs. Further information about the importance of these and similar structural properties, such as tunnels and cavities (33) and physicochemical properties of amino acids in the promiscuous active sites (i.e. pka shifts) (32), will be considered for further inclusion in the next version of ProtMiscuity.
